# An integrated DEA-fuzzy AHP method for prioritization of renewable energy sources in India

**DOI:** 10.1038/s41598-024-84891-2

**Published:** 2025-01-04

**Authors:** Jyoti Luhaniwal, Shivi Agarwal, Trilok Mathur

**Affiliations:** Department of Mathematics, Birla Institute of Technology and Science Pilani, Pilani Campus, Pilani, Rajasthan 333031 India

**Keywords:** Energy science and technology, Mathematics and computing

## Abstract

As India’s population grows and urbanization accelerates, energy demand is increasing sharply while conventional sources fall behind. To tackle energy shortages and climate change, India must prioritize renewable energy sources (RES), which offer sustainable solutions. The country is rich in RES, which can enhance fuel mix for electricity generation. This study analyzes various RES in India-solar, geothermal, hydro, biomass, wave, onshore, and offshore wind energy -using an integrated data envelopment analysis (DEA) and fuzzy analytic hierarchy process (Fuzzy AHP) methodology. Four main parameters-technical, economic, environmental, and socio-political -are identified and supported by 19 criteria, with environmental parameters including both desirable and undesirable criteria. In first phase, undesirable criteria are transformed into desirable criteria using Modified Ratio model. DEA is then applied to calculate initial efficiency score of RES under each parameter category. Fuzzy AHP determines weights for each parameter. The weights and initial efficiency scores are then combined to calculate overall efficiency score and ranking of RES. Sensitivity analysis shows that results obtained from proposed methodology are significant, and robust. Offshore wind ranks highest in efficiency, followed by hydro and onshore wind, while geothermal scores lowest. This methodology could benefit developing nations and guide policymakers in adopting RES.

## Introduction

Sustainable development has become crucial for the well-being of both society and nations. A widely cited and globally embraced definition of sustainable development is the notion of progress that satisfies the current generation’s requirements without harming the potential of future generations^1–3^. Energy is a critical component of sustainable development, driving economic growth and improving living standards. In India, industrialization and technological advancements have led to a significant increase in energy demand. As a result, the decline in fossil fuel reserves has caused economic burdens, social and political conflicts, and technological challenges. To address current and future energy needs, it is crucial to explore alternative energy sources. Renewable energy sources (RES) present a viable and sustainable solution to this challenge.

As a developing country, India has experienced substantial social progress and economic growth, leading to rising energy demands. This trend is expected to continue, supported by government initiatives like the Saubhagya Scheme and 24*7 Power for All. Additionally, policies such as Make in India and Atmanirbhar India, along with the adoption of e-mobility, will further increase energy demand^4,5^. India’s installed power capacity has expanded dramatically, from 1,713 MW in 1950 to 426,131 MW by 2023, representing an average growth rate of about 10.2% per year. By 2050, the International Energy Agency (IEA) estimates that India will require an additional power capacity of 600 GW to 1,200 GW^6^. As living standards improve and urban areas grow, India’s energy sector faces mounting pressures, including supply shortages, greenhouse gas emissions, and the need for extensive land for energy infrastructure. To overcome energy shortages, it is important to make good use of the plentiful renewable resources at hand. India has a range of RES, such as solar, hydro, biomass, onshore and offshore wind, wave, and geothermal energy. These energy sources can effectively produce electricity, address energy gaps, and support the country’s sustainable development. As of December 2023, India’s energy mix includes 56% thermal energy, 11% hydro, 2% nuclear, and 31% renewable sources^7^. Reducing the high share of non-renewable energy is essential to ensure long-term sustainability and energy security. India’s commitment to achieving net-zero emissions by 2070 highlights the necessity of transitioning to renewable energy^8^. Embracing RES not only meets energy needs but also drives economic growth, creates jobs, and helps reduce greenhouse gas emissions^9^.

Prioritizing various RES based on sustainability criteria is a complex challenge due to multiple dimensions. The 2002 World Summit on Sustainable Development identified social, environmental, and economic dimensions as key aspects for sustainability assessment^3^. Factors such as managing infrastructure, land use, and source variability complicate the decision-making process for optimal energy sourcing in India. Despite extensive research on ranking and selection of RES, several important gaps remain. First, many existing studies fail to address both desirable and undesirable outputs, particularly regarding environmental impacts. This study bridges that gap by converting undesirable outputs into desirable ones using the modified Ratio model and evaluated the efficiency of all RES utilizing the Data Envelopment Analysis (DEA) method, a method rarely applied in this field. Second, there is often little consideration for uncertainty in expert judgments, which can affect decision-making. This research incorporates Fuzzy Analytic Hierarchy Process (Fuzzy AHP) to manage such uncertainty, improving the accuracy of the results. Third, previous work tends to focus on limited criteria, such as technical and economic aspects, without considering the full range of factors. This study includes technical, economic, environmental, and socio-political factors, offering a more comprehensive evaluation. Furthermore, in the Indian context, no research has been conducted that considers wave and offshore wind energy as alternatives in the evaluation despite their significant potential. This study fills that gap by prioritizing these sources, making the findings more relevant to India’s energy landscape.

To address these gaps, this study uses an integrated DEA and Fuzzy AHP methodology. The DEA component and modified Ratio model address undesirable criteria by transforming them into desirable ones, after which efficiency scores are evaluated. Meanwhile, Fuzzy AHP manages uncertainties in expert assessments and determines the weights for the criteria. By incorporating a broader set of criteria-technical, economic, environmental, and socio-political-this research offers a more thorough evaluation of RES. Additionally, including wave and offshore wind energy expands the focus to less-explored renewable sources, providing valuable insights for India’s energy planning. The current study integrates DEA with Fuzzy AHP to improve the ranking process and provide a more effective solution for India. DEA is a flexible, non-parametric method that does not assume a predefined mathematical model for the data, making it suitable for analyzing diverse data types. It allows for simultaneous evaluation of multiple inputs and outputs without requiring predefined weights, ensuring objective assessment. DEA can handle both desirable and undesirable outputs, making it particularly useful for evaluating renewable energy sources by considering both benefits and impacts. By comparing each option against the best performers, DEA calculates efficiency scores, provides clear rankings, and identifies areas for improvement through benchmarking less efficient options against the most efficient ones. This method manages slacks more accurately and maintains both radial attributes and slack monotonicity. This ensures optimal utilization of all inputs and outputs when assessing the performance of renewable energy sources. These features make DEA a comprehensive and unbiased tool for assessing renewable energy sources. Fuzzy AHP, on the other hand, simplifies complex decision-making by organizing criteria into a clear hierarchy and prioritizing options based on expert judgments. Using fuzzy numbers accounts for the inherent vagueness in human preferences, effectively handling uncertainties. This study employs triangular fuzzy numbers to represent the imprecision inherent in human preferences, offering a balance between computational simplicity and the ability to model uncertainty effectively. Compared to other MCDM methods like TOPSIS and PROMETHEE, which often require precise data, Fuzzy AHP is particularly adept at dealing with imprecise information and managing multiple criteria. This makes it an accurate and adaptable choice for decision-making, especially when expert opinions and uncertainties are crucial. By combining DEA for efficiency analysis and Fuzzy AHP for incorporating expert insights, this integrated approach ensures more accurate, comprehensive, and balanced decision-making.

The remainder of this paper is organized as follows: “The application of MCDM in evaluating renewable energy sources” section offers a literature review on decision-making related to renewable energy. “Alternatives and parameters for evaluation of renewable energy sources (RES)” section presents an overview of available RES and parameters for RES evaluation. “Research framework” section outlines the proposed methodology. “Results” section presents the findings, sensitivity analysis, and implications for policy and management. Finally, “Conclusions” section 6 provides conclusions and recommendations based on the study’s results.

## The application of MCDM in evaluating renewable energy sources

Various studies have employed multi-criteria decision-making (MCDM) methods to prioritize RES. These methods are particularly suitable because selecting the optimal RES involves multiple, complex criteria that must be evaluated. Kongar and Rosentrater^10^ proposed a new approach employing DEA to analyze and rank RES based on efficiency and effectiveness. By considering various inputs and outputs, the model offered a thorough analysis of energy sources, aiding decision-makers in selecting the most suitable technology. The study addressed the growing demand for sustainable energy solutions amid increasing global energy needs and environmental concerns. Jha et al.^11^ concentrated on ranking renewable energy alternatives in India, utilizing Fuzzy AHP and an Energy Index parameter. This index was derived by aggregating scores across various alternatives, assessed based on eleven environmental and techno-economic criteria. Saglam ^12^ extensively examined the effectiveness of seven key RES using DEA, providing valuable insights into their comparative efficacy. By employing DEA methodologies, the research offered policymakers a robust framework to assess and prioritize RES based on their effectiveness and performance. Solangi et al.^13^ applied AHP and Fuzzy VIKOR methods to assess various sites for solar energy plants. Weights for each criterion and sub-criteria were obtained by AHP, and Fuzzy VIKOR performed a ranking of feasible alternatives. Xu et al.^14^ developed an integrated framework that combined Slack-Based DEA, Fuzzy AHP, and Fuzzy TOPSIS to rank and prioritize the most efficient and sustainable hydrogen production methods in Pakistan. In a recent study, Kolagar et al.^15^ introduced a hybrid methodology that merges DEA with the fuzzy best-worst method (FBWM) to prioritize RES in Iran. This method encompassed five sustainability facets: economic, technical, political, social, and environmental. Furthermore, a sensitivity analysis conducted in three stages validated the reliability of the DEA-FBWM approach compared to alternative decision-making approaches. In a study by Shah et al.^16^, the feasibility of various RES for hydrogen production in Pakistan was examined using a combination of Fuzzy Delphi, Fuzzy AHP, and environmental DEA methods. The researchers focused on six RES options: wind, solar, biomass, municipal solid waste, geothermal, and micro-hydro. First, Fuzzy Delphi was applied to establish the criteria and sub-criteria, followed by fuzzy AHP to calculate the relative weights of the criteria for selecting the best RES. Environmental DEA was then used to measure the efficiency of each RES, with the criteria weights serving as outputs and the cost of electricity generation as the input. The results indicated that wind energy emerged as Pakistan’s most efficient hydrogen production source. Solangi et al.^17^ developed a mathematical framework that combined AHP and Fuzzy TOPSIS to analyze barriers to renewable energy in Pakistan. The study first utilized the AHP method to rank the barriers and sub-barriers, identifying “Economic & Financial,” “Political & Policy,” and “Market” as the key challenges. Following this, the Fuzzy TOPSIS method evaluated strategies for the effective implementation of renewable energy technologies, indicating that “Capital Subsidies,” “Feed-in Tariffs,” and “Direct, Enabling, & Integrating Policies” were the most viable solutions to overcome these barriers. Shah et al.^18^ examined the energy crisis in Pakistan, where citizens face extensive power outages. The study employed the Fuzzy Delphi method to identify barriers to renewable energy deployment and then used the Grey AHP to assess the relative importance of these barriers across various RES. The study found that solar energy faced the fewest barriers, ranking highest. At the same time, policy and regulatory challenges emerged as the most significant obstacles to advancing renewable energy in the country. Longsheng et al.^19^ conducted a study where SWOT factors and strategies were finalized through a literature review, and FAHP was used to assign weights to these sub-factors based on input from fifteen anonymous experts in the energy sector. Grey-TOPSIS then employed the FAHP weights to prioritize twelve strategies derived from the SWOT analysis, offering a systematic approach to strategy evaluation. The given literature provides adequate evidence that the DEA and Fuzzy AHP methods are proficient in deciding on the selection and prioritization of RES. Therefore, this study applied the integrated DEA-Fuzzy AHP method to choose the most optimum RES for the sustainable production of electricity in India. Table 1 summarizes studies that rank RES in different countries using various MCDM methods. These methods assess and prioritize RES based on technical, economic, environmental, and socio-political parameters. This overview provides insights into how different approaches prioritize RES based on each country’s unique energy needs and goals. Table 2 highlights various case studies that evaluate RES in India using MCDM approaches. As shown in this table, two studies have also employed the Fuzzy AHP method to rank RES in the country.

**Table 1:** Ranking of RES by country and methodology.

Author	Country	Methods	Results
^20^	Turkey	Fuzzy AHP	Wind > solar > biomass > geothermal > hydro
		Fuzzy axiomatic design	
^11^	India	Fuzzy AHP	Geothermal > hydro > wind > biomass > solar
^21^	India	Shannon entropy	Solar > wind > hydro > biomass > nuclear > thermal
		Fuzzy AHP	
^22^	Chicago	AHP	Solar > wind > biomass > hydro > geothermal > nuclear
		VIKOR	
^23^	Turkey	IF-EDAS	Solar > hydro > wind > geothermal > hydrogen > biomass > wave
^24^	India	Entropy	Entropy: solar > wind > biomass > hydro
		CRITIC	CRITIC: solar > biomass > wind > hydro
^25^	China	AHP	Hydro > wind > biomass > solar > nuclear
		TOPSIS	
^26^	India	PF-VIKOR	Wind > hydro > biomass > geothermal > solar
^27^	Turkey	ANP	Hydro > solar > wind > geothermal > biomass
^28^	Turkey	IF-TOPSIS	Hydro > geothermal > wind > solar
^29^	Saudi Arabia	BWM	Gas > solar > hydro > coal > diesel
		TOPSIS	
^30^	Turkey	Fuzzy COPRAS	Wind > biomass > solar > geothermal > hydro
		Fuzzy MULTIMOORA	
^31^	Algeria	AHP	Solar > wind > biomass > geothermal > hydro
^32^	Turkey	Shannon Entropy	Hydro > geothermal > regulator > wind
		Fuzzy TOPSIS	
^16^	Pakistan	DEA	Wind > micro-hydro > solar > municipal solid waste > biomass > geothermal
		Fuzzy Delphi	
		Fuzzy AHP

**Table 2:** Indian case study in RES selection and ranking using MCDM methods.

Author	Year	Research purpose	Method	Results
^11^	2017	Assessment and prioritization of RES in terms of energy index	Fuzzy AHP	Geothermal is most preferred, followed by hydro, wind, biomass, and solar
^26^	2020	Evaluation of RES	PF-VIKOR	Wind is identified as highly suitable source, followed by hydro, biomass, geothermal and solar
^21^	2021	Evaluation of energy alternatives for sustainable development of energy sector	Shannon entropy	Economic and environmental are most important criteria and solar and wind are identified as top suitable, while gas power, thermal, and nuclear energy are least preferred
			Fuzzy AHP	
^24^	2024	Selection of an optimal RES	Entropy	Solar to be the best source for energy production, followed by wind and biomass
			CRITIC	
^33^	2024	Selection of best RES	Fuzzy GTMA	Wind energy is the most preferred among primary RESs due to its lower cost, mature technology, and negative impact on the environment, followed by solar and biomass energy

Building on the above-mentioned research gaps and previous work, the current paper aims to integrate the DEA method with Fuzzy AHP to enhance the ranking process and provide a more effective solution for India. DEA is a non-parametric approach, meaning it does not assume a mathematical form for the data, giving it flexibility in handling different data types. It allows the analysis of multiple inputs and outputs at the same time. Unlike other methods, DEA does not need predefined weights for inputs and outputs, making the evaluation more objective. It can also handle both desirable and undesirable outputs, which is helpful when considering the benefits and impacts of renewable energy sources. It evaluates efficiency scores by comparing each option to the best performers, allowing for clear rankings and helping identify areas where improvements are needed by benchmarking less efficient options against the best ones. These features make DEA an effective tool for evaluating RES comprehensively and unbiasedly. Fuzzy AHP breaks down complex problems into a clear hierarchy of criteria, making it easier to analyze and prioritize different options. By considering subjective expert judgments through fuzzy numbers, this method captures the vagueness of human preferences. Compared to other MCDM methods, Fuzzy AHP excels at managing uncertainty and dealing with multiple criteria. While methods like TOPSIS and PROMETHEE often require exact measurements, Fuzzy AHP effectively handles imprecise information. Overall, it provides a flexible and accurate way to make decisions, especially when expert opinions and uncertainties are significant. DEA provides an efficiency analysis in this integrated method, while Fuzzy AHP incorporates expert knowledge, allowing for more accurate and well-rounded decision-making. This study contributes to the literature by addressing the limitations of conventional DEA models, such as CCR and BCC, which often struggle with slack handling and the full utilization of input and output criteria. By employing the New Slack Model (NSM) DEA, this research introduces a method that accurately manages slacks while maintaining radial attributes and slack monotonicity, ensuring the efficient utilization of inputs and outputs. Furthermore, the integration of the NSM model with the Modified Ratio model offers a novel solution for converting undesirable criteria into desirable outputs, a gap not thoroughly explored in previous studies. The inclusion of fuzzy AHP enhances the robustness of the analysis by effectively assigning parameter weights and addressing uncertainties in expert judgments. This comprehensive approach, encompassing criteria handling, weight assignment, efficiency evaluation, and ranking, represents a significant advancement, offering a more holistic and adaptable framework for prioritizing renewable energy sources.

## Alternatives and parameters for evaluation of renewable energy sources (RES)

India’s economy has experienced significant growth, yet the energy supply still needs to match this accelerated growth. Consequently, there has been a substantial increase in India’s energy demand^34^.

**Fig. 1:**
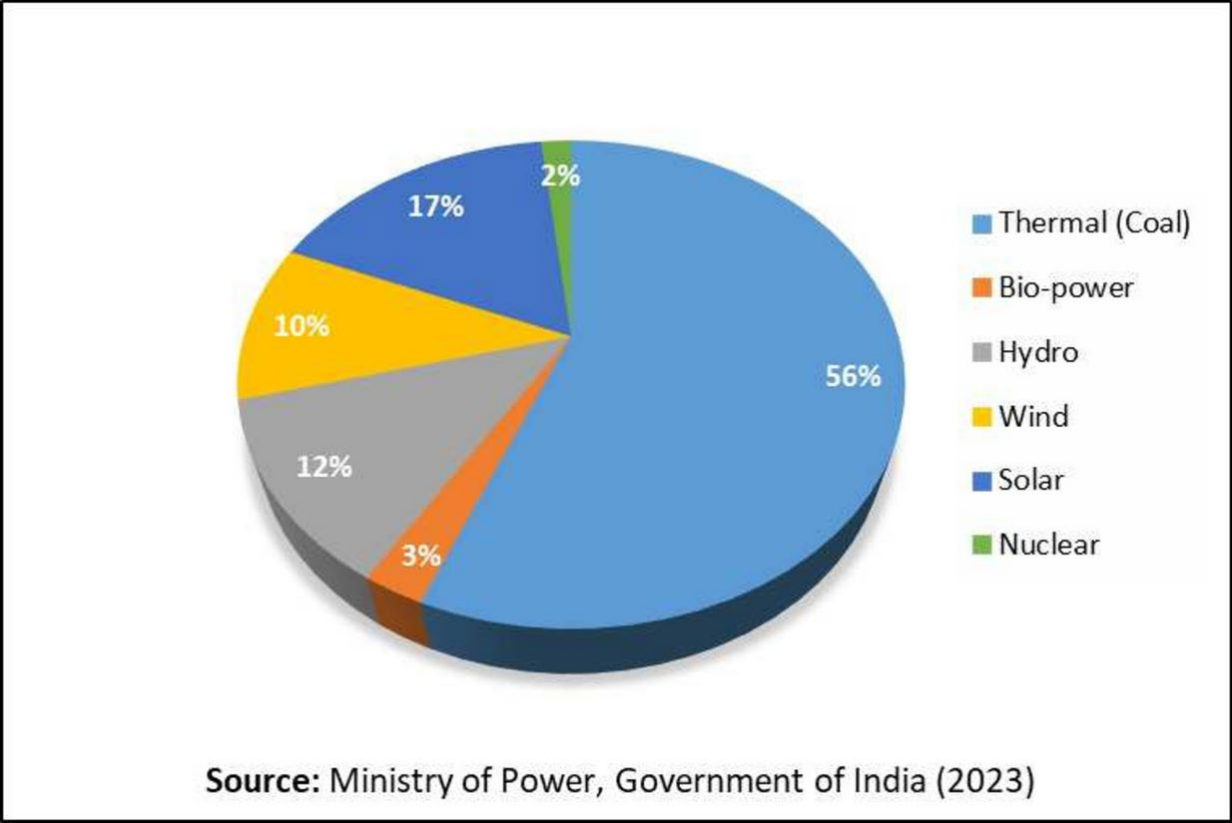
Installed power capacity of India.

India, situated in the northern hemisphere, spans from the latitudes $8^{\circ }40^\prime$ to $37^{\circ }6^\prime$ North to longitudes $68^{\circ }7^\prime$ and $97^{\circ }25^\prime$ East, making it one of the largest countries in Asia. With a land area of approximately 3.287 million $\text {Km}^2$, India experiences rapid population growth and urbanization. Consequently, India’s energy demand has steadily risen in recent years. In 2022, India’s total energy consumption is around 650 Mtoe, projected to escalate to 1200 Mtoe by 2030^35^. As of December 2023, India’s installed capacity consists of 56% thermal energy, 11% hydro energy, 2% nuclear energy, and 31% renewable energy sources, as shown in Fig. 1^7^. The Indian government is focused on boosting renewable energy, strengthening this sector, and promoting its development through various initiatives. Despite India’s rapid economic growth, the energy supply has failed to keep pace, resulting in a substantial increase in the country’s energy demand^34^. The rise in population and environmental concerns highlight the urgent need for sustainable energy, making renewable sources a priority for researchers. Renewable sources replenish more rapidly than they are consumed^36^. Globally, India is fourth in installed renewable energy capacity, encompassing large hydro, and ranks fourth in solar and wind energy capacity^37^. A brief overview of the RES is described in the below section.

### Solar energy

Solar power stands out as the limitless and plentiful resource of renewable primary energy, freely accessible to all. The efficient harnessing of solar radiation reaching Earth annually could produce energy equivalent to 10,000 times the global energy demand for a year^38^. India’s location amidst the Tropic of Cancer and the Equator results in an average yearly temperature of 25 $^{\circ }$C to 27.5 $^{\circ }$C and significant solar radiation, ranging from 1200 kWh/m^2^/year to 2300 kWh/m^2^/year. Within the solar belt, India receives about 3000 sun hours yearly, equivalent to a power of 5000 trillion kWh. This advantageous positioning underscores India’s substantial solar energy potential. The Jawaharlal Nehru National Solar Mission (JNNSM), initiated by the government of India in 2010 through the National Action Plan on Climate Change (NAPCC), focuses on accelerating solar energy generation by fostering and advancing solar resources in India to attain 2000 MW of off-grid solar power and 20,000 MW of grid solar power^39^. Although solar energy is commonly regarded as a highly sustainable technology for energy generation, emissions are associated with it to some extent due to the use of substances such as mercury and cadmium in fabricating solar cells^40^.

### Hydro energy

Hydroelectric power plants convert water’s potential energy into electricity, offering a sustainable energy solution that harnesses the Earth’s natural water cycle. These facilities provide benefits such as quick response to peak load demands, fast on/off capabilities, and energy storage to fulfill daily, seasonal, and peak demand needs^41^. India holds a significant hydroelectric potential, placing it fifth worldwide for its harnessable hydroelectric potential. India’s primary hydroelectric power station, featuring a 130 kW capacity, commenced operations in Darjeeling in 1879^42^. India’s hydraulic energy installation capacity is increasing rapidly, with the installed capacity reaching approximately 39,788.40 MW as of September 30, 2013, and currently standing at 46,512 MW as of 2022^43^. Hydroelectric power projects entail significant time and financial investment. India still has opportunities for further expanding its hydropower generation, as the development of mini- and micro-hydropower systems could be viable alternatives for harnessing the nation’s water resources and channels to generate cost-effective electricity from hydroelectric power^44^. While they emit fewer greenhouse gases, NOx, and SO$_2$, they disrupt socio-environmental systems by altering agricultural and irrigation practices, land procurement, environmental clearance procedures, and community resettlement^45^.

### Biomass energy

India, focusing on agriculture, possesses abundant biomass resources, including agricultural residue, animal waste, and organic components from municipal and industrial waste^46^. Diverse technologies enable the conversion of biomass into usable energy. India’s substantial biomass power potential includes 17,538 MW from agricultural residue, 5000 MW from the combined bagasse generation, and 2556 MW from organic and municipal waste^11^. Biomass has historically served as a significant energy source for the nation due to its environmentally friendly nature, plentiful supply, renewability, and capacity to create employment opportunities in rural areas. Furthermore, biomass contributes to sustainable energy production. The primary drivers for biomass-to-energy conversion in India revolve around cost reduction and enhanced efficiency^47^. Across India, facilities for biomass energy production, including those for both bagasse and non-bagasse processing, have been established, collectively generating 10,205.61 MW of electricity for the grid.^7^. Gujarat, Madhya Pradesh, Rajasthan, and Punjab are the top states in biomass energy production. Biomass energy has ecological drawbacks, such as emitting greenhouse gases and minor emissions of $NO_x$ and $SO_x$^40^.

### Geothermal energy

Geothermal energy, sourced from heat retained within the Earth’s crust and evident on the surface through phenomena such as hot springs and geysers, is prominent in countries like the USA, Indonesia, Philippines, Turkey, and New Zealand. In India, the Geological Survey of India (GSI) has approximated a theoretical potential of 10 GW that could be harnessed from geothermal energy^48^. This established and dependable renewable energy source holds considerable potential in India. Geothermal energy proves versatile and operates effectively in both connected-to-the-grid and off-grid setups. It offers significant benefits in countryside power supply initiatives. It serves diverse needs like industrial operations, space heating, warmth provision, greenhouse heating, aquatic activities, bathing, agricultural drying, and various other applications^49^. The exploration of geothermal energy in India commenced in 1862. The Geological Survey of India has surveyed 340 thermal springs and identified several promising geothermal regions, including the Puga Valley in Ladakh district, which stands out as particularly prospective. These assessments indicate a substantial geothermal potential of 10,600 MW, yet much remains untapped in practice^50^.

### Onshore wind energy

Onshore wind power energy can provide electricity for both residential and industrial purposes, representing a mature and cost-effective technology with a well-established supply chain^51^. The generation process involves transforming the kinetic energy of moving air using wind turbines, which arises from the airflow phenomenon resulting from the uneven heating of the Earth’s outer layer. Widely recognized as a renewable energy option, onshore wind power reduces dependence on petroleum and other fossil fuels. By the end of 2023, India had installed nearly 44.96 GW of onshore wind capacity, poised to significantly contribute to the country’s electricity objectives^52^. Across elevations spanning from 20 to 120 m, 794 specialized platforms have been constructed to monitor wind conditions, aiming to assess the potential of wind energy^7^.

### Offshore wind energy

Offshore wind can generate electricity on an industrial scale. On a global scale, offshore wind has a history spanning approximately two decades, beginning with Denmark’s installation of the first offshore wind turbine in 1991, which was decommissioned in 2017. Offshore wind power projects have a combined capacity exceeding 18 countries. The foremost nations include the UK, the Netherlands, Germany, China, and Denmark. Currently, India has not installed any offshore wind capacity. Nevertheless, the initial evaluation of offshore wind energy potential within designated zones suggests approximately 70 GW off the coasts of Tamil Nadu and Gujarat^7^. Consequently, offshore wind is anticipated to bolster India’s renewable energy objectives significantly. Offshore wind turbines, typically ranging between 5 to 10 MW per turbine, are more significant than their onshore counterparts, which usually produce between 2 to 3 MW per turbine. While offshore turbines incur higher costs per MW due to the need for robust structures and foundations in the marine environment, the potential for advantageous tariffs arises from the improved efficiencies of these turbines as the ecosystem matures^7^.

### Wave energy

Wave energy is extracted from the agitation of water surfaces caused by winds interacting with the surface of the sea or ocean. It is typically captured from waves with average heights ranging between 2 and 3 meters. Wave energy is deemed more efficient than solar and wind energy as an unconventional energy source. However, its widespread use is limited due to the high costs associated with energy conversion^30^. According to a survey conducted in December 2014, the projected theoretical capacities for wave energy are estimated at 41,300 MW^53^. Wave energy generation depends on both the amplitude of the wave and its duration. Initial assessments of the potential for harnessing wave energy along India’s coastline suggest a range of approximately 5–15 MW per meter, resulting in a theoretical estimated potential of around 40–60 GW.

### Parameters and criteria

After identifying the RES for comparison and prioritization, the next step is to establish the parameters and criteria for assessing these sources. The selection of parameters and criteria varies by country, influenced by specific conditions, energy goals, and development strategies. This study selects the criteria by reviewing many studies from India and other countries to identify widely recognized and relevant factors. The widely recognized criteria in these studies are adopted to ensure they are relevant and easy to compare. This approach ensures that the selected criteria align with earlier research and provide a strong basis for the analysis. The number of criteria chosen is also based on the availability of quantitative and qualitative data that are pertinent, accessible, and capable of producing meaningful results. Previous research has highlighted a variety of factors that support sustainable energy development. In this study, these factors were grouped into four main parameters: technical, economic, environmental, and socio-political. A total of 19 criteria are selected from an extensive literature review within these categories. Each criterion can be classified as either desirable or undesirable, and this classification plays an important role in evaluating the alternatives. The selected criteria, along with their associated parameters and sources, are summarized in the following sections and presented in Table 3.

**Table 3:** Parameters and criteria of the decision model.

Parameters	Criteria	References
Technical	C1: Deployment time [years]	^15,54^
	C2: Technical maturity	^24,55^
	C3: Efficiency	^21,24^
	C4: Capacity factor	^11,24^
Economic	C5: Capital cost [USD/kW]	^21,56^
	C6: Operation and maintenance cost [USD/MW]	^15,54^
	C7: Payback period [years]	^33^
	C8: Levelized cost of electricity [USD/kWh]	^24,55^
	C9: Operational life [years]	^21^
Environmental	C10: Land requirement [$\text {km}^2$/1000 MW]	^54–56^
	C11: $CO_2$ emission [mg/Kwh]	^11^
	C12: $SO_2$ emission [mg/Kwh]	^11^
	C13: $NO_x$ emission [mg/Kwh]	^11^
	C14: Impact on ecosystem	^33^
Socio-political	C15: Foreign dependency	^21,33^
	C16: Job creation [jobs/500 MW]	^33,54^
	C17: Social acceptance [%]	^11,21^
	C18: Political acceptance [%]	^21,55^
	C19: Social benefits	^33,54^

#### Technical parameter

- *Deployment time (C1)* refers to the duration required for the entire process of planning, designing, constructing, and commissioning the power plant until it becomes operational and starts generating electricity.
- *Technical maturity (C2)* signifies the extent to which a particular technology is adopted and utilized on regional, national, and global scales. It also indicates whether the technology has already achieved its maximum theoretical efficiency or if there are opportunities for further enhancements.
- *Efficiency (C3)* serves as a frequently utilized technical measure for evaluating energy systems, signifying the amount of sound energy that can be obtained from an energy source. The efficiency coefficient, commonly employed as a measure of efficiency, is described as the proportion of output energy to input energy.
- *Capacity Factor (C4)* of a power plant is determined by the ratio of the actual electricity generated during a specific timeframe to the potential electricity that could have been produced if the plant had been operating at its maximum power output throughout that period.

#### Economic parameter

- *Capital cost (C5)* encompasses the complete expenditure involved in establishing a plant, which includes costs associated with equipment, labor, installation, infrastructure, and commissioning.
- *Operations & maintenance costs (C6)* encompass the expenses associated with the day-to-day operation of a plant, including employee salaries, costs of parts and spares required for scheduled maintenance, and other related expenditures. While O&M costs for renewable energy plants are generally lower when compared to fossil fuel-fired power plants, they still represent a substantial portion of the overall expenses.
- *Payback period (C7)* of an energy project represents the time frame needed for the returns on an investment to equal or exceed the initial investment amount.
- *Levelized Cost of Electricity (C8)* stands for the cost of energy generated per unit. It is expressed in *Rs*/*kWh* or $\$/kWh$.
- *Operational life (C9)*, or service life, of a power plant refers to the duration it can effectively operate before decommissioning. This lifespan is typically measured in years.

#### Environmental parameter

- *Land requirement (C10)* for power generation holds an opportunity cost affecting human habitation, utilization, and environment. For a fair comparison of technologies, this study analyzes land use across power plant lifecycles, encompassing fuel extraction, processing, transport, waste disposal, construction, operation, and decommissioning.
- $CO_2$ *emission (C11)* plays a vital role in the greenhouse effect. A substantial portion of it is released into the atmosphere during the combustion of fossil fuels for electricity generation.
- $SO_2$ *emission (C12)* is one of the detrimental gases released into the atmosphere during electricity generation, posing a significant risk to power plant workers.
- $NO_X$ *emission (C13)* Similar to $SO_2$ emission, this poisonous gas is discharged into the atmosphere during power generation. Its presence exerts negative implications on the environment, contributing to environmental degradation and posing potential risks to the ecosystem.
- * Impact on the ecosystem (C14)* is defined as the change in the local ecosystem due to the introduction of RE technology.

#### Socio-political parameter

- *Foreign Dependency (C15)* mainly analyzes the dependency of fuel imports from foreign countries.
- *Job creation (C16)* refers to the capacity of an energy project to generate potential employment opportunities. Renewable energy development should consider the impact on local residents by evaluating improvements in quality of life and the potential for job creation.
- *Social acceptance (C17)* is crucial for adopting renewable energy technologies and achieving energy policy goals, gauging public approval of RE. It’s pivotal as public and advocacy group opinions can impact project timelines.
- *Political acceptance (C18)* involves acknowledging and endorsing renewable energy sources by government policies concerning technological advancement. It encompasses the alignment of RES with political, legislative, and administrative frameworks, ensuring their compliance with existing regulations and governance structures.
- *Social benefits (C19)* refer to the positive impact and advancement within the local community and region resulting from initiating a power project.

**Fig. 2:**
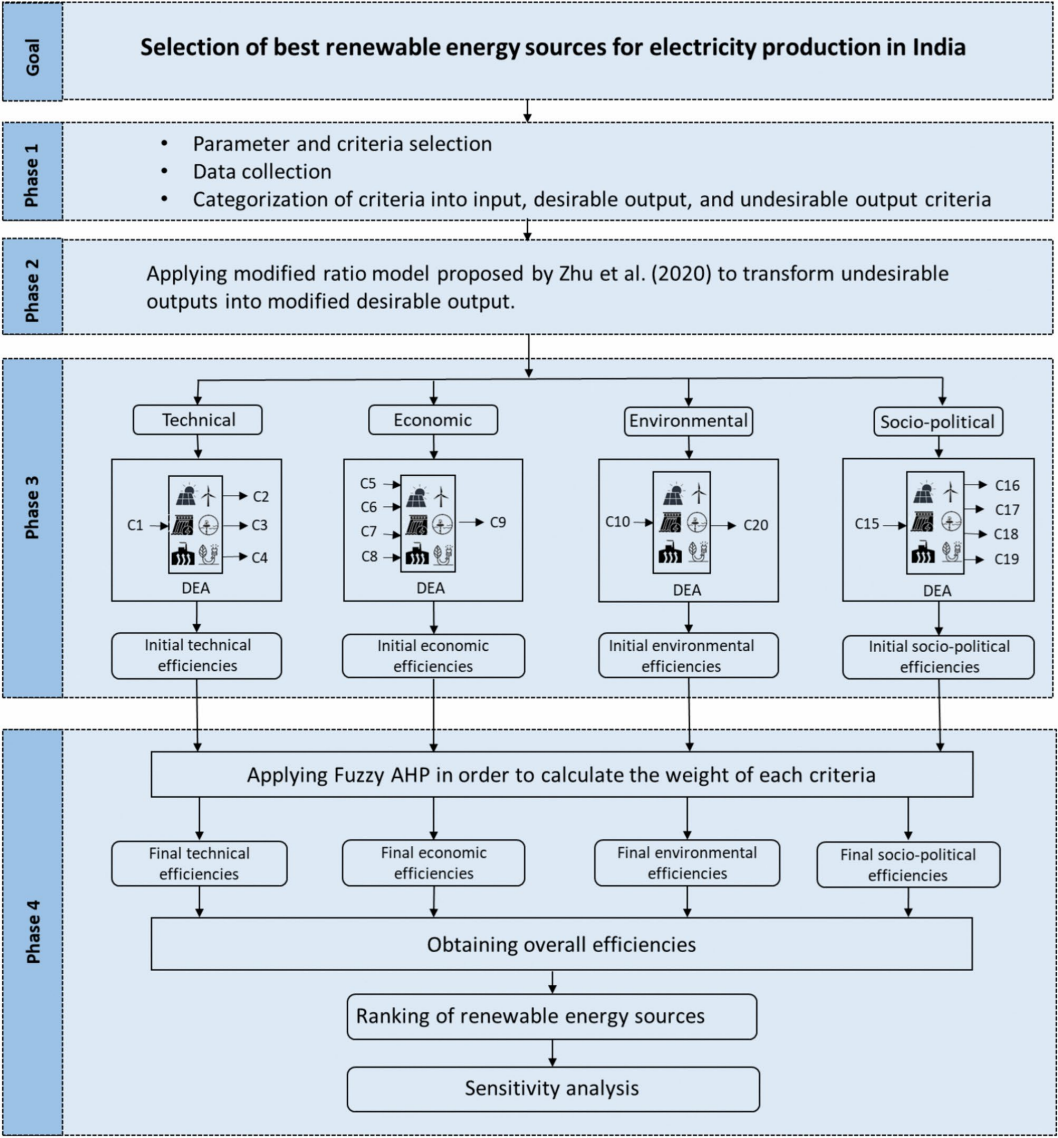
Research framework.

## Research framework

To fulfill the objective of this study, the research framework is divided into four phases. In the first phase, parameters and criteria for evaluating RES for electricity generation are identified through a comprehensive literature review using Science Direct, Google Scholar, Web of Science, and Scopus databases. At this stage, which criteria will be treated as input, desirable output, and undesirable output criteria are also determined. Data is gathered from various secondary sources, including annual reports, balance sheets of India’s renewable energy organizations, literature reviews, and expert inputs^37,57–60^. Since RES can also produce undesirable outputs, this study addresses these by using a modified ratio model. In the second phase of the analysis, the model proposed by Zhu et al. (2020) is applied to transform undesirable output criteria into desirable ones. This transformation ensures a more accurate and comprehensive evaluation of RES. In the third phase, after transforming these undesirable criteria, the data is prepared for applying the NSM-VRS output-oriented DEA model to calculate the initial efficiencies for each of the four sustainability parameters across the seven RES. In the final phase, Fuzzy AHP is used to calculate the weights for each sustainability parameter, using expert inputs to ensure accurate assessments. This study employs triangular fuzzy numbers to represent the imprecision inherent in human preferences, offering a balance between computational simplicity and the ability to model uncertainty effectively. These weights are then combined with the initial efficiency scores to derive the overall efficiency score and establish the relative ranking of the RES. As the final ranking of alternatives heavily relies on expert weighting, a sensitivity analysis based on criteria weights is performed to demonstrate the stability of the ranking. For this, various scenarios are examined, each involving different parameter weight allocations. A schematic diagram of the research framework is presented in Fig. 2.

### Methods

#### Modified ratio model proposed by Zhu et al.^61^

Consider *R* Decision Making Units (DMUs) denoted as $(r=1,2, \ldots , R)$ which produce *n* outputs using *m* inputs $X_{ik}(i=1,2, \ldots , m)$. Among these outputs, $n_1$ are classified as undesirable (denoted as $\text{B}_q^{-}(q=1,2, \ldots , n_1)$), and $n_2$ are categorized as desirable (denoted as $Y_p^{+}(p=1,2, \ldots , n_2)$), with $n_1+n_2=n$. Given our ultimate objective of merging undesirable outputs with desirable ones, desirable outputs can, to some extent, show the input data of DMUs. Consequently, when computing the combined weights of undesirable outputs, inputs are excluded, and only the desirable outputs $Y_p^{+}$ and undesirable outputs $B_q^{-}$are considered. This computation occurs in two stages, as outlined below. Initially, the efficiency scores of DMUs are determined utilizing the output-oriented multiplier model. These scores are obtained by considering the desirable and undesirable outputs as inputs within the model.

The model is :

1 $$\begin{aligned} \begin{aligned}&\min \delta _k=\sum _{q=1}^{n_1} v_{q k} b_{q k} \\ \text{ s.t. }&\sum _{p=1}^{n_2} u_{p k} y_{p k}=1, \\&\sum _{p=1}^{n_2} u_{p r} y_{p r}-\sum _{q=1}^{n_1} v_{q r} b_{q r} \le 0, ~~~r=1,2, \ldots , R, \\&v_{q r} \ge 0, ~~~q=1,2, \ldots , n_1, \\&u_{p r} \ge 0, ~~~p=1,2, \ldots , n_2. \end{aligned} \end{aligned}$$

In this context, $v_q$ and $u_p$ symbolize the weights allocated to the undesirable and desirable outputs, respectively, while $\delta$ indicates the efficiency score of the assessed DMU, ranging from 1 to infinity. An efficiency score of 1 indicates that the evaluated DMU is efficient. The efficiency score attained for each DMU using this model is represented as $\delta ^*$. It can be applied in the subsequent step, which involves determining the aggregate weights $\rho _q$ of the various undesirable outputs $B_q^{-}$ using equation (3).

2 $$\begin{aligned} \begin{aligned}&\min _{n_1} \alpha \\ \text{ s.t. }&\sum _{q=1}^{n_1} \rho _q b_{q r}-\delta _r^* \le \alpha , \\&\sum _{p=1}^{n_2} \rho _{p r} y_{p r}=1, \\&\sum _{q=1}^{n_1} \rho _q b_{q r}-\delta _r^* \ge 0, ~~~r=1,2, \ldots , R, \\&\rho _q \ge 0, ~~~q=1,2, \ldots , n_1, \\&\rho _{p r} \ge 0, ~~~p=1,2, \ldots , n_2. \end{aligned} \end{aligned}$$

Here, $\delta ^*$ denotes the efficiency score derived from model (1); $\rho _q$ represents the aggregate weight of an undesirable output; $\rho _{pr}$ indicates the weight of a desirable output; and $\alpha$ signifies the maximum deviation between the efficiency score of the multiplier model and the weighted undesirable outputs. The objective function aims to minimize $\alpha$. Desirable outputs are bound by $\sum _{p=1}^{n_2} \rho _{pr} y_{pr}=1$. The weights assigned to the desirable outputs $\left( \rho _{pr}\right)$ are individual weights, and these constraints do not impact the values of the aggregate weights of undesirable outputs obtained from model (2). In essence, these constraints are unnecessary Importantly, the aggregate weights obtained from model (2) lack dimensions, thus evading issues related to the attributes of the original measures. $\Psi _k$ denotes the aggregated undesirable outputs, which can be evaluated as:

3 $$\begin{aligned} \begin{aligned} \Psi _k=\rho _1 B_{1 k}^{-}+\ldots +\rho _{n_1} B_{n_1 k}^{-}. \end{aligned} \end{aligned}$$

Here, $\rho _q\left( q=1,2, \ldots , n_1\right)$ represents the aggregate weights of undesirable outputs. The modified desirable output $Y_p^{\prime }$ is obtained by dividing $\Psi _k$ by $Y_p^{+}$, where $\Psi _k$ serves as the denominator and $Y_p^{+}$ as the numerator:

4 $$\begin{aligned} \begin{aligned} Y_p^{\prime }=\frac{1}{\Psi _k} Y_p^{+},\left( p=1,2, \ldots , n_2\right) . \end{aligned} \end{aligned}$$

Here, $Y_p^{\prime }$ can encompass attributes of both desirable and undesirable outputs concurrently. Eventually, the efficiency scores of all DMUs can be ascertained using the conventional DEA model (5).

#### Data envelopment analysis (DEA)

DEA is widely regarded as a powerful non-parametric method for measuring performance and efficiency, offering advantages over traditional methods like ratio analysis and regression. Unlike these approaches, DEA does not require a predefined production function, giving it flexibility to handle different types of data. DEA uses linear programming to calculate the relative efficiency of DMUs, allowing them to select the best combination of inputs and outputs to maximize efficiency. This flexibility has led to its extensive use in various fields, such as banking, supply chain management^62^, and energy and environmental assessments^63^. DEA creates an efficiency frontier composed of the most efficient DMUs and compares all other units against this frontier. Units on the frontier are deemed efficient, while those below it are considered inefficient. DEA identifies the gap between inefficient DMUs and the frontier, offering insight into where improvements can be made. This is particularly useful when handling multiple inputs and outputs, as DEA compares each DMU to the best performers. The original DEA model, known as the CCR model, was introduced by Charnes, Cooper, and Rhodes in 1978, assuming constant returns to scale. Later, in 1984, Banker, Cooper, and Charnes developed the BCC model, which incorporated variable returns to scale by adding a convexity constraint. This made DEA more adaptable to varied scenarios. Further advancements included the Cone Ratio Model^62^, Slack Adjusted Model^64^, Slack-Based Model^63^, and the New Slack Model (NSM)^65^. This study employs the New Slack Model (NSM) to address the limitations of traditional DEA models. Unlike CCR and BCC models, which do not handle slacks well, and SBM model lacking radial characteristics, NSM maintains both radial attributes and slack monotonicity. This ensures all inputs and outputs are fully utilized when assessing DMU performance. The output-oriented NSM-VRS model applied here provides a more thorough analysis, ensuring all multipliers are positive, thereby improving the overall assessment of DMU efficiency.

5 $$\begin{aligned} \begin{aligned}&\operatorname {Max} \bar{\theta }_k= \theta _k+\frac{1}{m+n_2}\left( \sum _{p=1}^{n_2} \frac{s_{p k}^{+}}{y^{\prime }_{p k}}+\sum _{i=1}^m \frac{s_{i k}^{-}}{x_{i k}}\right) \\&\text{ subject } \text{ to } \\&\sum _{r=1}^R \lambda _{r k} y^{\prime }_{p r}-s_{p k}^{+}=\theta _k y^{\prime }_{p k} \quad \forall p=1,2, \ldots , n_2, \\&\sum _{r=1}^R \lambda _{r k} x_{i r}+s_{i k}^{-}=x_{i k} \quad \forall i=1,2, \ldots , m, \\&\sum _{r=1}^R \lambda _{r k}=1,\\&\lambda _{r k}\ge 0, \quad \forall r=1,2, \ldots , R\\&s_{i k}^{-}, s_{p k}^{+} \ge 0, \quad \forall i=1,2, \ldots , m;~~ p=1,2, \ldots , n_2.\\&\end{aligned} \end{aligned}$$

where $\lambda _{r k}$ represents the dual variable corresponding to the $r^{th}$ constraint and is termed as the intensity variable, $s^+_{pk}$ signifies the slack in the $p^{th}$ output of the $k^{th}$ DMU, $s^-_{ik}$ indicates the slack in the $i^{th}$ input of the $k^{th}$ DMU. $x_{ik}$ denotes the observed quantity of the $i^{th}$ input of the $k^{th}$ DMU, $y^{\prime }_{pk}$ denotes the observed quantity of the $p^{th}$ modified output of the $k^{th}$ DMU. $\theta _k$, unrestricted in sign, represents the proportional adjustment employed to all outputs of the $k^{th}$ DMU to enhance efficiency.

#### Fuzzy AHP

The initial work on Fuzzy AHP was introduced by Van Laarhoven and Pedrycz (1983)^66^, integrating AHP with fuzzy set theory. This approach is particularly effective in managing the uncertainty present in decision-making processes. Decision-makers often prefer to provide subjective or interval judgments rather than fixed values, as they may find it difficult to precisely express their preferences due to the inherent vagueness in the comparison process. FAHP organizes the problem into a hierarchical structure consisting of a goal, criteria, and sub-criteria, making it easier to systematically evaluate options. The fuzzy AHP method employs pairwise comparisons to allocate weights to criteria and rank various energy alternatives using Table 4. Opinions from both academic and renewable energy experts are collected through a questionnaire utilizing linguistic terms, with the aim of applying the Fuzzy AHP method. Questionnaire are carried out in accordance with relevant guidelines and regulations and confirming that informed consent is obtained from all subjects and/or legal guardian(s). Experimental protocol are approved by institutional committee.

Step - I. Research conducted using the fuzzy AHP method introduced by Buckley in 1985^67^.

Step - II. The relative significance of sustainable energy alternatives is determined through pairwise comparisons. The weights are computed using the fuzzy geometric mean method, with equations derived from Saraswat et al. (2021)^21^.

6 $$\begin{aligned} a=\left( p_1 \times p_2 \times \ldots \times p_n\right) , b=\left( q_1 \times q_2 \times \ldots \times q_n\right) , c=\left( r_1 \times r_2 \times \ldots \times r_n\right) \end{aligned}$$

*n* denotes count of criteria.

Step - III. Fuzzy weights are derived by multiplying the fuzzy geometric mean and the reciprocal of the sum of those fuzzy geometric mean values, as detailed in equation (6).

7 $$\begin{aligned} \begin{aligned}&w_l=a_l\left( a_1+a_2+\ldots +a_n\right) ^{-1}, w_m=a_m\left( a_1+a_2+\ldots +a_n\right) ^{-1}, \\&w_u=a_u\left( a_1+a_2+\ldots +a_n\right) ^{-1} \end{aligned} \end{aligned}$$

Step - IV. De-fuzzified crisp numeric values (DCNV) are determined by averaging the fuzzy lower, medium, and upper values, as specified in equation (7)..

8 $$\begin{aligned} D C N V=\frac{w_l+w_m+w_u}{3} \end{aligned}$$

Step - V. To validate the consistency of the expert’s opinion, the consistency ratio is assessed. A consistency ratio below 0.1 indicates an accurate determination of criteria weight.

9 $$\begin{aligned} C R=\frac{C I}{R I} \end{aligned}$$

10 $$\begin{aligned} C I=\left( \lambda _{\max }-n\right) /(n-1) \end{aligned}$$

where, *CI* represents the consistency index, *RI* denotes the random index, $\lambda _{\max }$ signifies the maximum eigenvalue, and *n* stands for the number of criteria.

**Table 4:** Fuzzy AHP Scale.

Linguistic statement	Fuzzy-AHP scale
Equally important	(1,1,1)
Weakly important	(2,3,4)
Fairly important	(4,5,6)
Strongly important	(6,7,8)
Absolutely important	(9,9,9)
	(1,2,3)
	(3,4,5)
	(5,6,7)
Interpolation scale	(7,8,9)

## Results

India boasts various RES, including biomass, solar, geothermal, wind, hydro, and wave. Harnessing electricity from these renewable sources underscores India’s significant potential to generate power from sustainable fuels.

Table 3 lists the sustainable parameters and all the nineteen criteria falling under these parameters. The results of implementing the proposed integrated DEA-Fuzzy AHP method to evaluate the prioritization of renewable energy alternatives with a focus on sustainable development are outlined in this section.

### Selection of parameter, criteria and data colletion

The study begins by identifying various RES in India and establishing evaluation criteria through an extensive literature review and expert consultation. A decision-making team of nine experts from academia and renewable energy organizations in India is formed. In phase I, parameters related to sustainability assessment and criteria for each parameter are identified through literature review, authoritative articles, books, and expert consultation. The literature review and expert insights identify prominent RES: Solar, Offshore Wind, Onshore Wind, Biomass, Hydro, Wave, and Geothermal Energy. These alternatives are evaluated using economic, technical, socio-political, and environmental parameters within the sustainable development framework. This research considers these parameters for a robust evaluation of renewable energy sustainability, ensuring effective oversight of the energy sector. Following the selection of sustainability parameters, criteria are listed under these parameters. It is then determined which criteria served as inputs and desirable and undesirable outputs in the DEA model for each parameter. Accordingly, the inputs and outputs are identified as follows: C1 as the input and C2, C3, and C4 as the outputs for the technical parameter, C5, C6, C7, and C8 as inputs and C9 as the output for the economic parameter, C10 as the input and C11, C12, C13 are undesirable outputs, and C14 as the desirable output for the environmental parameter, and finally C15 as the input and C16, C17, C18, and C19 as the outputs for the socio-political parameter. Additionally, the inputs and outputs for each category are visually represented in the second phase of Fig. 2. To conduct the data envelopment analysis, data from various secondary sources have been collected. Quantitative data regarding to criteria C1, C4, C5, C6, C7, C8, C9, C10, C11, C12, C13, C14, and C16 are extracted from multiple annual reports and balance sheets of India’s renewable energy organizations^37,57–60^. Data pertaining to criteria C2, C3, C17, and C18 is gathered from extensive literature, while the remaining data for C15 and C19 is based on opinions from the decision-making team, assessed on a 10-point scale as depicted in Table 5. In practical scenarios, qualitative data often needs to be incorporated into the structure of data envelopment analysis. All the statistical data is presented in Table 6.

**Table 5:** Ten-points scale scoring the criteria.

Scale	Description
2	Compared to the other sources, its level is extremely low
4	Compared to the other sources, its level is low
6	Compared to the other sources, its level is medium
8	Compared to the other sources, its level is high
10	Compared to the other sources, its level is extremely high
1, 3, 5, 7, 9: intermediate values are used to compromise between two judgments.

**Table 6:** Research data statistics for implementing data envelopment analysis.

Criteria	Mean	SD	Min	Max
C1	3.036	2.324	0.500	7.000
C2	37.429	13.988	20.000	57.000
C3	39.114	27.106	9.500	97.300
C4	43.714	21.110	15.000	77.000
C5	1934.429	1224.018	22.000	3991.000
C6	1.478	2.824	0.021	8.360
C7	3.420	3.316	0.390	10.000
C8	0.060	0.013	0.045	0.080
C9	26.429	6.389	20.000	40.000
C10	843.571	1715.174	1.000	5000.000
C11	30.634	15.998	13.000	58.000
C12	109.143	102.721	10.000	280.000
C13	268.571	441.341	10.000	1325.000
C14	62.319	43.884	16.131	149.400
C15	3.143	1.125	2.000	5.000
C16	11531.943	9388.176	2500.000	27050.000
C17	63.714	12.532	40.000	80.000
C18	63.714	12.532	40.000	80.000
C19	6.286	1.485	4.000	9.000

### Modified ratio model results

In light of the increasing emphasis on environmental performance and sustainable production practices, the study first applies the modified ratio model proposed by Zhu et al. (2020)^61^. In Phase II, the modified ratio model is used to convert undesirable outputs related to environmental factors, such as $CO_2$, $SO_2$, and $NO_x$ emissions, into desirable outputs. This step is important for aligning these outputs with the sustainability goals of the study. First, efficiency scores are calculated using model (1), where the undesirable outputs are treated as inputs, and the positive impact on the ecosystem (C14) is treated as the desirable output. These efficiency scores, identified as $\delta ^*$, are subsequently applied in the model (2) to ascertain the aggregate weights for the various undesirable outputs. After calculating the aggregate weights, as shown in Table 7, the study computes $\Psi _k$, which represents the total undesirable outputs, using equation (3). Next, the modified desirable output $Y_p^{\prime }$ is calculated by combining both the desirable and undesirable outputs into one value through equation (4). This creates a modified desirable output for the environmental parameter, now called C20. This approach ensures that undesirable outputs are effectively included in the evaluation, helping provide a more complete assessment of renewable energy sources.

**Table 7:** Aggregate weights $\rho _q$ of the undesirable outputs.

$\varvec{\rho _1}$	$\varvec{\rho _2}$	$\varvec{\rho _3}$
$7.3704999\times 10^{-2}$	$1.389881\times 10^{-2}$	$8.79374\times 10^{-3}$

### DEA initial efficiency results

After computing the modified desirable output, the data is organized to apply the NSM-VRS output-oriented DEA model. In phase III, this model is used to calculate the initial efficiency scores for each of the four sustainability parameters across the seven RES. These calculations are carried out using MATLAB version R2020b software, and the results are presented in Table 8, showing the initial efficiency scores for each RES under different parameters. In the technical parameter, hydro, biomass, and wave energy show a high efficiency score of 1, indicating they are fully efficient in this category. Onshore wind has a slightly lower efficiency score of 0.913, meaning it performs well but not as efficiently as the top performers. Offshore wind, solar, and geothermal have lower scores of 0.758, 0.681, and 0.491, respectively, showing room for improvement in technical efficiency. For the economic parameter, two of the seven RES exhibit inefficiencies, with hydro and geothermal scoring 0.895 and 0.526, respectively, while the remaining RES display efficient economic performance. Regarding the environmental parameter, offshore wind and hydro achieve full efficiency with scores of 1, whereas biomass performs poorly with an efficiency score of 0.019, indicating significant inefficiencies in its environmental impact. In the socio-political parameter, solar energy demonstrates full efficiency with a score of 1, while biomass and offshore wind follow with scores of 0.809 and 0.805, respectively. Hydro, however, reflects lower efficiency in this parameter, with a score of 0.491.

**Table 8:** Initial efficiency scores.

Alternatives	Technical	Economic	Environmental	Socio-political
Solar	0.681	1.000	0.228	1.000
Onshore Wind	0.913	1.000	0.740	0.574
Offshore Wind	0.758	1.000	1.000	0.805
Hydro	1.000	0.895	1.000	0.491
Biomass	1.000	1.000	0.019	0.809
Geothermal	0.491	0.526	0.466	0.756
Wave	1.000	1.000	0.266	0.577

### Fuzzy AHP results and ranking of RES

In phase IV, opinions from both academic and renewable energy experts are collected through a questionnaire utilizing linguistic terms, with the aim of applying the Fuzzy AHP method using Table 4 in the first week of March month. Fifteen experts from academia, renewable energy, and environmental sectors were reached out, and nine of them agreed to participate in the survey. While some studies have also used four or ten experts, choosing nine qualified experts in this study balances diverse perspectives with a focused analysis^16,68,69^. Responses were collected virtually for the questionnaire in the first week of March month, spanning approximately 1.1 months. Respondents were requested to provide pairwise comparisons for the evaluation criteria using the relative importance attributes scale for the Fuzzy AHP method outlined in Table 4. Following the collection of responses for pairwise comparison of four sustainable parameters, we analyzed them to determine the weights for each of these parameters. These terms are subsequently converted into triangular fuzzy numbers (TFNs) using equations (6) and (7) to determine the weight of each sustainability parameter. The model is then solved in MS-Excel to derive optimal TFN values $(l_1, m_1, u_1),~(l_2, m_2, u_2),~(l_3, m_3, u_3),$ and $(l_4, m_4, u_4)$. These TFN values are further transformed into crisp values using equation (8), enabling the calculation of the optimal weights for the sustainability parameters. Finally, the outcomes of the model solution, along with the overall weights of the sustainability parameters, are presented in Table 9. Given that the CR value of 0.078, below the threshold of 0.10 in Fuzzy AHP, indicates high consistency and stability in the comparisons used to calculate the weights of sustainability parameters. Table 9 illustrates that economic parameter possesses higher weights, surpassing the importance of the remaining factors with a weight share of 43.9%, while the environmental parameter hold relatively lower weights accounting for 25.6%. Moreover, the technical parameter holds a relative weight of 19.2%, that is the third most important parameter. Socio-political parameter is given relatively less emphasis in terms of weights accounting for 11.3%. Consequently, it can be inferred that investors should prioritize economic and environmental parameter when selecting most appropriate renewable energy sources to advance sustainability layouts. In the last step, using the weights derived from Fuzzy AHP method, we obtained the efficiencies associated with each sustainability parameter, calculated by multiplying the initial efficiency score and corresponding weights for each of parameter. Consequently, the efficiencies are averaged to determine the overall efficiencies and rankings of each renewable energy source, as illustrated in Table 10. The ranking of energy sources using the DEA-Fuzzy AHP method is as follows: Offshore Wind > Hydro > Onshore Wind > Wave > Solar > Biomass > Geothermal. These rankings are based on the higher overall efficiency scores, with Offshore Wind Energy emerging as the most suitable renewable energy source for India’s future, boasting the highest overall efficiency value of 0.932 among the available sources. Hydro energy achieved the second rank with an efficiency score of 0.896, followed by onshore wind energy in third place with an efficiency of 0.869. Wave, solar, biomass, and geothermal energy obtained the fourth, fifth, sixth, and seventh ranks respectively, as shown in Table 10.

**Table 9:** Weights for criteria.

Criteria	TFNs	Value	Overall weight
Technical	$l_1$	0.102	0.192
	$m_1$	0.194	
	$u_1$	0.383	
Economic	$l_2$	0.256	0.439
	$m_2$	0.451	
	$u_2$	0.846	
Environmental	$l_3$	0.111	0.256
	$m_3$	0.260	
	$u_3$	0.533	
Socio-political	$l_4$	0.061	0.113
	$m_4$	0.116	
	$u_4$	0.222

**Table 10:** Overall efficiencies and rank of the renewable energy alternatives.

Alternatives	Technical	Economic	Environmental	Socio-political	Overall efficiencies	Rank
Solar	0.131	0.439	0.058	0.113	0.741	5
Onshore wind	0.175	0.439	0.190	0.065	0.869	3
Offshore wind	0.146	0.439	0.256	0.091	0.932	1
Hydro	0.192	0.393	0.256	0.055	0.896	2
Biomass	0.192	0.439	0.005	0.091	0.727	6
Geothermal	0.094	0.231	0.119	0.085	0.530	7
Wave	0.192	0.439	0.068	0.065	0.764	4

## Sensitivity analysis

**Fig. 3:**
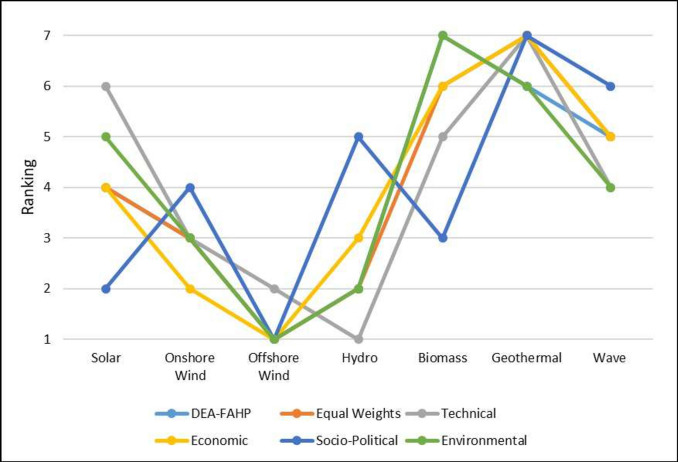
RES ranking for different weight-changing scenarios.

As the final ranking of alternatives heavily relies on expert weighting, a sensitivity analysis based on criteria weights is performed in this section to demonstrate the stability of the ranking. To achieve this objective, various scenarios are examined, each involving different weight allocations for the parameters. These weight variations are based on the five scenarios outlined in Table 11. This analysis aims to explore the impact of parameter weight adjustments on the final rankings. These scenarios, labeled from I to V, allocate weights differently. In Scenario I, all parameters are equally weighted. Scenario II assigns 50% weight to the technical parameter, distributing the remaining 50% among the economic, environmental, and socio-political parameters. Scenarios III to V individually prioritize the economic, environmental, and socio-political parameters as shown in Table 11. This approach allows us to assess how varying degrees of importance assigned to each dimension influence the final rankings. The weights for specific criteria are determined by dividing the dimension weight by the number of criteria. Table 12 and the accompanying chart in Fig. 3 illustrate the rankings under different scenarios. Notably, rankings vary across scenarios. In Scenario I, offshore wind emerges as the most favorable option, while geothermal is deemed the least attractive. The probable explanation could be its economic viability, strong social acceptance, relative environmental friendliness, political approval, and promotional efforts. From a technical standpoint, hydro exhibits the most promising performance, followed by offshore wind, onshore wind, wave, biomass, solar, and geothermal. Hydro is given preference due to higher technical maturity and higher efficiency. In terms of economic considerations, offshore wind remains the preferred choice. Offshore wind energy is favored for its low initial capital investment, shorter payback period, and minimal levelized cost of electricity. In Scenario IV, with an emphasis on maximizing environmental impact, offshore wind power emerges as the top choice, followed by hydro. This is primarily due to its lack of land requirement, which helps preserve terrestrial ecosystems and minimal pollutant emissions. Similarly, in Scenario V, offshore wind remains the preferred option, with solar as the next favorable choice and geothermal ranked the least desirable from this perspective. This preference could be attributed to its widespread social acceptance and advantages, minimal adverse effects on human health, and its capacity for generating employment opportunities at the community level. The rankings across various scenarios consistently favor offshore wind due to its economic viability, socio-political agreement, and environmental benefits. Considering the overall results obtained from the multiple sensitivity analysis scenarios in this study, the proposed DEA-Fuzzy AHP approach consistently adapts to changes in criteria formulation, making it the most reliable method. Therefore, it can efficiently evaluate and prioritize RES. However, the suitability of renewable energy sources varies based on specific criteria, emphasizing the importance of a detailed evaluation approach.

**Table 11:** Criteria weight for different cases.

Parameter	Scenario-I	Scenario-II	Scenario-III	Scenario-IV	Scenario-V
	Equal weights	Technical	Economic	Environmental	Socio-political
Technical	0.25	0.500	0.167	0.167	0.167
Economic	0.25	0.167	0.500	0.167	0.167
Environmental	0.25	0.167	0.167	0.500	0.167
Socio-political	0.25	0.167	0.167	0.167	0.500

**Table 12:** Sensitivity analysis results.

Alternatives	DEA-Fuzzy AHP	Equal weights	Technical	Economic	Environmental	Socio-political
Solar	5	4	6	4	5	2
Onshore Wind	3	3	3	2	3	4
Offshore Wind	1	1	2	1	1	1
Hydro	2	2	1	3	2	5
Biomass	6	6	5	6	7	3
Geothermal	7	7	7	7	6	7
Wave	4	5	4	5	4	6

## Discussion

This study assesses the sustainability of seven RES in India using an integrated DEA-Fuzzy AHP model that evaluates technical, economic, environmental, and socio-political parameters. In Phase I, criteria are selected through a literature review and classified into input, desirable, and undesirable outputs. Phase II transforms undesirable criteria into desirable ones using the modified Ratio model. Phase III applies DEA for initial efficiency calculations, followed by Fuzzy-AHP for parameter weighting in Phase IV. The RES are ranked based on overall efficiencies, with economic parameters receiving the highest weight (0.439) and socio-political the lowest (0.113). Offshore wind, hydro, and onshore wind rank as the top three, while geothermal and biomass rank lower. Sensitivity analysis confirms offshore wind’s consistent top ranking, enhancing model reliability. The ranking of energy sources using the DEA-Fuzzy AHP method is as follows: Offshore Wind > Hydro > Onshore Wind > Wave > Solar > Biomass > Geothermal. In this study, offshore wind energy is found to be the highest-ranking RES in terms of efficiency, and the next are hydro and onshore wind aligning with recent research that highlights its potential for high performance and scalability. However, our findings contrast with some existing studies in India that prioritize solar energy or hydropower. The contrast arises because previous studies in India did not include alternatives like offshore wind energy and wave energy, which can significantly influence the prioritization of energy sources. Since the DEA model operates on a relative efficiency basis, offshore wind emerges as a more suitable option compared to solar and hydro energy when evaluated within this framework. A comparison of parameter weights with previous studies demonstrates strong alignment, with only slight variations. These differences can be attributed to the specific criteria chosen and regional factors that influence the prioritization of renewable energy sources. Additionally, when comparing the criteria and parameters used in our study with those in similar research, it is evident that while many studies emphasize economic and technical factors, our inclusion of socio-political and environmental criteria provides a more comprehensive assessment. This broader approach enhances the depth of the evaluation, addressing a wider range of challenges in renewable energy decision-making. The application of an integrated DEA-Fuzzy AHP approach is effective in addressing undesirable and conflicting criteria, providing a more robust evaluation framework compared to traditional standalone AHP or DEA models. Unlike Kolagar et al.^15^, environmental factors are divided into desirable and undesirable criteria, and further undesirable criteria are modified into desirable ones. Moreover, none of the studies in India included wave energy and offshore wind despite having significant potential. Governments should revise policies favoring conventional fuels and technologies, giving priority to renewable energy sources like offshore wind, hydro, and onshore wind for sustainability. India must accelerate the development of these energy sources to address power shortages, mitigate climate change, and reduce global warming. By focusing on offshore wind for its scalability, hydro for its reliability, and onshore wind for its accessibility, India can lead the fight against climate change while benefiting its environment, economy, and society. This study makes several significant contributions to the existing literature based on the discussion.

## Policy and decisional implications

The findings of this study offer significant insights for policymakers, practitioners, and investors, emphasizing the prioritization of offshore wind, hydro, and onshore wind energy as the top renewable energy sources. These rankings align with India’s energy strategy and ongoing efforts to enhance renewable energy integration. Policymakers should prioritize offshore wind energy by streamlining regulatory processes, upgrading infrastructure, and providing financial incentives to attract investment. Current initiatives in Gujarat and Tamil Nadu have identified potential offshore wind zones, while policies aimed at harnessing hydroelectric resources in the North and North-Eastern regions reflect the government’s commitment to maximizing renewable energy potential. Addressing challenges like variable water availability, environmental concerns, and infrastructure needs is vital. Effective policies must focus on improving storage systems and ensuring project sustainability to optimize hydro’s contribution to the energy mix. Onshore wind farms, with their established infrastructure, can complement offshore projects by providing consistent energy output, particularly in regions with favorable wind conditions. By leveraging the study’s results, policymakers can allocate resources more effectively to enhance energy security and meet sustainability targets. A stronger focus on renewable energy also contributes to reducing carbon emissions and achieving net-zero goals. For investors, the study provides a clear roadmap for prioritizing investments in efficient and sustainable energy projects. Emphasizing top-ranked sources, such as offshore wind, minimizes risks and ensures optimal returns while supporting the country’s transition to a resilient energy system. The study’s structured approach to evaluating renewable energy sources can inform the design of policies that promote a diversified energy mix. This ensures long-term sustainability, economic growth, and environmental benefits, aligning with India’s national energy objectives.

## Conclusions

Renewable energy is essential for meeting India’s rising energy needs while combating climate change and reducing dependence on fossil fuels. This study prioritized RES in India, addressing key gaps by incorporating undesirable criteria using the modified Ratio model, overcoming a common oversight in prior studies that treated undesirable factors as desirable, and evaluating efficiency through the NSM DEA model, which effectively handles slacks and maintains both radial attributes and slack monotonicity. This ensures optimal utilization of all inputs and outputs when assessing the performance of renewable energy sources. To account for the inherent uncertainty in expert judgment, the study employed Fuzzy MCDM, ensuring robustness in the analysis, while social, technical, environmental, political, and economic criteria provided a holistic assessment, including underexplored resources like offshore wind and wave energy. Although this study offers valuable insights, it is important to acknowledge certain limitations. The findings are specific to the Indian context, and applying the methodology to other regions may require adjustments to account for differences in local RES types and influencing factors. Additionally, the study employed triangular fuzzy numbers to address uncertainty and human judgment, while effective, could be refined with advanced fuzzy techniques such as trapezoidal or interval-type fuzzy numbers. Future research could enhance this framework by adopting advanced DEA models capable of handling undesirable factors with greater precision. Given India’s diverse climatic conditions and geography, conducting state-wise analyses would offer a deeper understanding of the most suitable RES for each region, contingent on the availability and quality of regional data. Furthermore, this methodology could be extended to assess renewable energy systems in other countries or applied to industries beyond the energy sector, thereby broadening its applicability and providing a more global perspective.

## Data Availability

The author confirms that all data generated or analyzed during this study are included in this manuscript.
